# Regional differences in multidimensional aspects of health: findings from the MRC cognitive function and ageing study

**DOI:** 10.1186/1471-2458-6-90

**Published:** 2006-04-06

**Authors:** Fiona E Matthews, Laura L Miller, Carol Brayne, Carol Jagger

**Affiliations:** 1 MRC Biostatistics Unit, Institute of Public Health, Robinson Way, Cambridge, CB2 2SR, UK; 2 Dept. Public Health and Primary Care, Institute of Public Health, Cambridge, UK; 3 Department of Health Sciences, University of Leicester, Leicester, UK; 4 For participants see

## Abstract

**Background:**

Differences in mortality and health experience across regions are well recognised and UK government policy aims to address this inequality. Methods combining life expectancy and health have concentrated on specific areas, such as self-perceived health and dementia. Few have looked within country or across different areas of health. Self-perceived health, self-perceived functional impairment and cognitive impairment are linked closely to survival, as well as quality of life.

This paper aims to describe regional differences in healthy life expectancy using a variety of states of health and wellbeing within the MRC Cognitive Function and Ageing Study (MRC CFAS).

**Methods:**

MRC CFAS is a population based study of health in 13,009 individuals aged 65 years and above in five centres using identical study methodology. The interviews included self-perceived health and measures of functional and cognitive impairment. Sullivan's method was used to combine prevalence rates for cognitive and functional impairment and life expectancy to produce expectation of life in various health states.

**Results:**

The prevalence of both cognitive and functional impairment increases with age and was higher in women than men, with marked centre variation in functional impairment (Newcastle and Gwynedd highest impairment). Newcastle had the shortest life expectancy of all the sites, Cambridgeshire and Oxford the longest. Centre differences in self-perceived health tended to mimic differences in life expectancy but this did not hold for cognitive or functional impairment.

**Conclusion:**

Self-perceived health does not show marked variation with age or sex, but does across centre even after adjustment for impairment burden. There is considerable centre variation in self-reported functional impairment but not cognitive impairment. Only variation in self-perceived health relates to the ranking of life expectancy. These data confirm that quite considerable differences in life experience exist across regions of the UK beyond basic life expectancy.

## Background

Describing and addressing health inequalities are a key focus for public health agendas in many countries of the world and highlighting inequalities at local area level, rather than through personal characteristics such as ethnicity or socio-economic status, are especially important in countries such as the UK where health care resources are devolved down to a local level. Most research on geographical variations in health has been based on mortality data, due to its accessibility and level of detail. Inequalities in mortality between local areas in the UK have been well documented from as early as 1841[1] and these still persist today,[2] with rural and more prosperous areas having the highest life expectancies,[3] though much of this may be accounted for by migration[4]. Furthermore the geographical variations in life expectancy appear to have widened between 1984 and 1994[5].

Over the last century, the ageing of populations has resulted in greater emphasis being given to the quality of remaining years (measured through morbidity) rather than the quantity (measured through mortality). Indeed, the relevance of health expectancies for monitoring health policy has been recognized by three government strategies in the UK [6-8] all aimed at improving healthy life expectancy based on self-perceived health, which itself has a strong correlation with service use and mortality, the latter over and above measures of physical health or disability [9,10].

Within the UK, the regional (local authority) variation in healthy life expectancy (as measured by limiting longstanding illness) has been found to be much greater than the regional variations in life expectancy (at birth, in men 6.5 years difference in healthy life expectancy versus 3 years difference in total life expectancy and in women 5 years versus 2.5 years, from the 1991 Census) with a strong north-south gradient[11] and this has been replicated for larger geographic areas (health authorities)[12]. Similar findings have been reported from other countries [13], specifically Canada (lower disease free life expectancy (DFLE) in rural and northern regions), France (north-south gradient but also distinct regional changes in rankings over time) and Spain (again striking gains and losses for specific regions over time). In Belgium the size of regional variations appears to depend on the health measure used with regional differences in DFLE being less important than differences in life expectancy[14] though the opposite was true for healthy life expectancy based on self-reported health[15]. Although in most countries inequalities in healthy life expectancy reflect those in life expectancy, in the Netherlands women's healthy life expectancy at age 65 was independent of life expectancy at age 65[16]. Some countries have sought to explain geographic variations in healthy life expectancy through area-level indicators of socio-economics and healthcare availability[11,16-19]. Often the institutional population has been omitted from calculations, a weakness in Western countries where institutional care for older people is common.

As health data at a regional level are sparse, the majority of the current literature has reported findings for a single measure of health, often DFLE, though definitions of disability vary widely, or healthy life expectancy based on self-perceived health. To obtain a more balanced view of overall health this paper presents a detailed comparison of a range of health expectancies, covering self-perceived health together with actual self-reported functional status and measured cognitive ability of older people aged 65 and above, including those in institutional care. Data have been taken from the MRC Cognitive Function and Ageing Study (MRC CFAS) based in five geographically diverse areas of England and Wales extending the previous analysis based on all centres combined[20]. The distributions of self reported functional impairment and cognitive measures have been fully described in previous papers[21,22].

## Methods

### Study design and population

The Medical Research Council Cognitive Function and Ageing Study (MRC CFAS) is a multi-centre prospective cohort study, and is community based including institutions. The study design (fully described elsewhere[22]) is based in six geographically defined areas, four urban (Liverpool, Newcastle, Nottingham, Oxford) and two rural (Cambridgeshire, Gwynedd). Liverpool was funded earlier than the other sites and, although comparable, followed a slightly different methodology and is not included in this paper as the outcomes of interest were not measured in the same way. The sites were chosen as representing the main national variation in urban-rural differences, regional heterogeneity, and the presence of research groups experienced in population studies. Ethical approval was obtained at each study site.

Stratified populations of people aged 65 and over, including those in institutions, were randomly selected from Family Health Service Authority lists to achieve an interviewed sample of approximately 2,500 people in each centre. The screening interview, used in this analysis, covered socio-demographic details, activities of daily living, self-reported physical health including risk factors for dementia, cognitive function and medication [22].

Interviews were carried out using laptop computers in the respondent's place of residence. Routing through the schedule was controlled by response to each question; inapplicable questions were not displayed. Interviewers were instructed to try to ask all questions wherever possible. For those who failed to show they were orientated in time and place, a shorter route was possible which offered only priority questions – the memory section of the Geriatric Mental state (GMS) [23], a short version of the cognitive section and interviewer ratings. This enabled calculation of Mini-Mental State Examination (MMSE) score[24] and the Automated Geriatric Examination Computer Assisted Taxonomy (AGECAT) organicity level only[25].

MRC CFAS has Multi-centre Research Ethics Committee approval and ethical approval from the relevant Local Research Ethics Committees. All participants gave informed consent. Data from the initial screen (Version 3.1 of the dataset) are used in this paper.

### Health domains

Two impairment areas were examined: functional (based on activities of daily living), and cognitive (based on MMSE score). Self-perceived health was measured using the question "Would you say that for someone of your age, your health in general is ... excellent/good/fair/poor" and compared between the five centres. We also examined the relationship between self-perceived health and two impairment areas.

The questions related to functional impairment were from the Townsend disability scale [26], which covers Activities of Daily Living and Instrumental Activities of Daily Living. The specific questions were "are you able to ... cut toe nails/wash all over/get on a bus/go up and down stairs/do heavy housework/carry heavy bags/prepare and cook a hot meal/reach an overhead shelf/tie a good knot in a piece of string". If individuals were unable to undertake the item they were asked whether they could undertake the activity with help. Each item was scored with 0 for able with no help, 1 for able with help and 2 for those unable. The total score is calculated from the sum of these 9 questions. Functional impairment was defined as being a score of 11 or more out of a maximum of 18, equating to standard methods and is equivalent to mild ADL impairment across all domains or severe impairment across five domains[26]. If an individual was bedbound or chairbound they were classified as having a functional impairment.

Cognitive impairment has been defined using the Mini-Mental State Examination (MMSE)[24]. The established cut-point of below 18 on the scale from 0–30 was used to identify severe cognitive impairment[27]. No adjustment has been made for age or education for this cut-point.

### Statistical methods

Health expectancies were calculated using Sullivan's method[28] with abridged life tables. The population and mortality data by age and sex in five year age groups from each of the five centres were obtained. Each of the five centres were defined by district geographical health areas; Cambridgeshire (East Cambridgeshire and Fenland); Gwynedd (Ynys Môn (Anglesey) and Dwyfor); Newcastle upon Tyne; Nottingham; Oxford. Population data from 1991 were obtained from the small area census volumes and mortality data for the same year from the Office for National Statistics[29,30].

Prevalences of each of the impairments by region were calculated. To explore whether regional differences in prevalence were simply a reflection of the burden of disease we fitted multivariable logistic regression models adjusted for age, sex, serious health problems and the other impairments together with region and examined the differences. Health problems have been examined using the number of health conditions reported (from stroke, angina, intermittent Claudication, chronic bronchitis, asthma (except in childhood), visual impairment, hearing impairment, diabetes, Parkinson's disease, high blood pressure (GP confirmed), depression, epilepsy and arthritis). To ensure that regional differences were not determined by educational effects on cognitive impairment, modelling of cognitive impairment was additionally adjusted for years of full-time education. The confidence intervals for the regional effects have been estimated using floating absolute risks[31]. This allows us to compare regions without the need for each comparison to be made via the reference category and is preferable where no natural referent exists. The model for self-perceived health was additionally investigated using ordered logistic regression and adjusts for age, sex, functional and cognitive impairment.

There was a variable amount of missing data for each of these measures. A sensitivity analysis was carried out with missing data assumed as indicating impairment. The data are not shown as this did not produce substantially different life expectancies from those shown.

## Results

13,009 individuals contributed to the prevalence screen with roughly equal numbers in each of the centres (Table 1). Despite the sampling strategy that oversampled those individuals age 75 and above, small numbers of the very old, particularly men can be seen.

**Figure 1:**
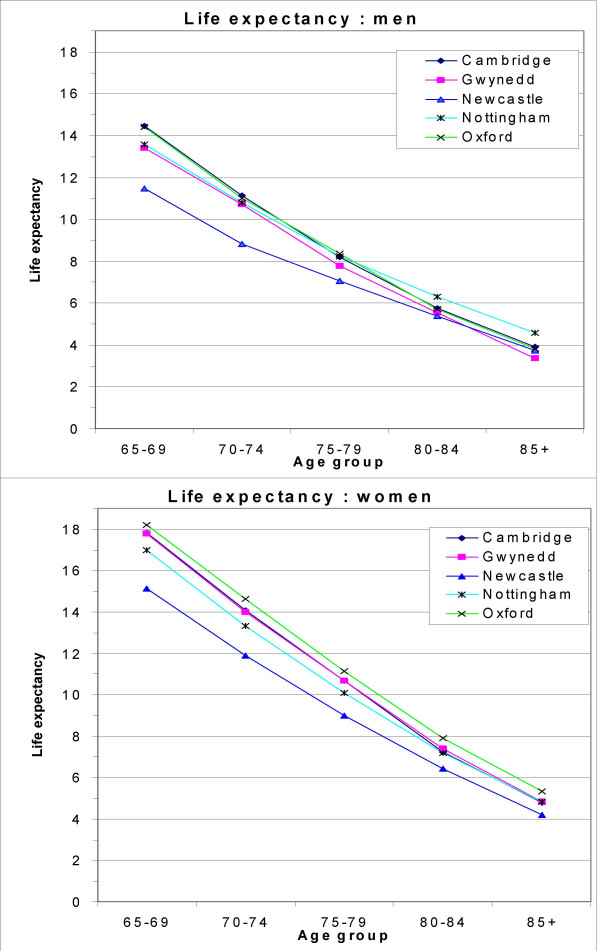
Life expectancy from age 65 years for men and women, by centre.

Life expectancies show distinct regional differences at all ages (Figure 1). Newcastle had the shortest life expectancy for both men and women at all ages. Cambridge and Oxford had the highest life expectancies in men though the life expectancies for Cambridge, Oxford and Gwynedd were similar in women. All centres appear to converge at extreme old age (Table 1).

The age- and sex-specific prevalence of health states and impairments by region are shown in Table 1. The proportion of individuals classifying themselves as in fair or poor health increases slightly with age, though above 85 years the increase is less marked or even reverses in both men and women and for all centres. Cambridgeshire and Oxford have a higher self-perceived health than the other centres. All centres showed similar effects by age and sex for the two impairment domains. Self reported functional impairment is more common than cognitive impairment at the cutpoints chosen. The absolute proportions with impairment will vary according to the chosen cut points.

Modelling the variation across centres in self-perceived health, functional impairment and cognitive impairment revealed marked differences in the first two, but not in the latter (Table 2). Patterns of self-perceived health do not appear to reflect just impairment burdens. Modelling self-perceived health revealed that it is strongly related to both functional and cognitive impairment but regional effects persist after adjusting for these other impairments (Table 2).

**Figure 2:**
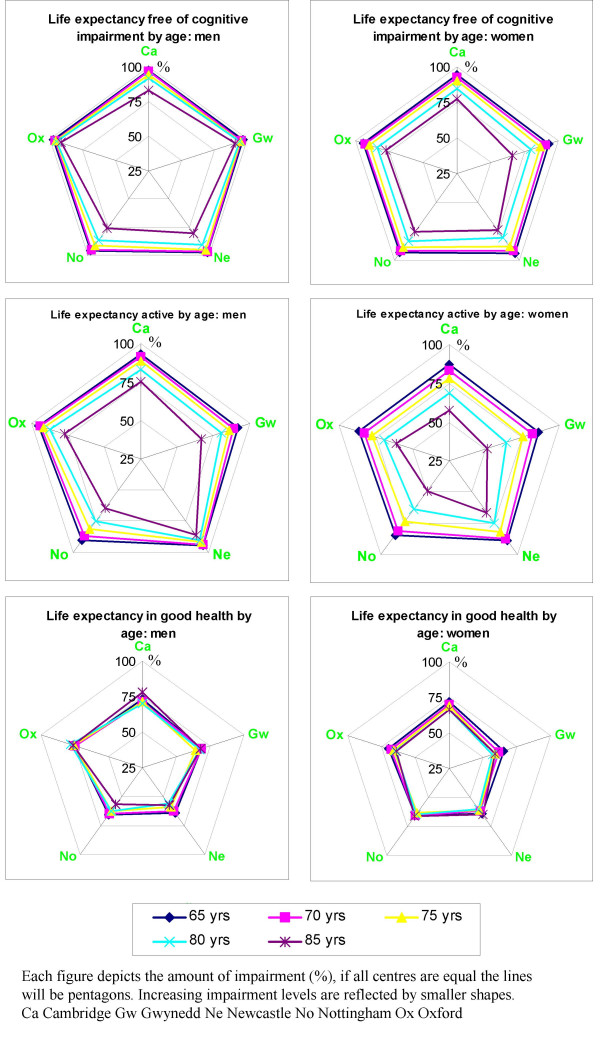
Proportion of remaining life spent active (free of functional impairment), in good self-perceived health and free of cognitive impairment by age and centre for men and women.

The proportion of life spent healthy, using each of the health domains, generally declines with age but there are large differences across centres (Figure 2). Each figure depicts the proportion of the total life expectancy with impairment by centre. If all centres are the same the lines would be perfect pentagons, the larger the impairment the smaller the shape. Nottingham had the lowest proportion of life spent active in men and women. For men Newcastle has the smallest variation across age in the proportion of remaining life spent healthy with men aged 85 and above spending 87% of remaining life active compared to 64% in Nottingham. Women in Gwynedd and Nottingham have the fastest decline in the proportion of life spent active across age, from 85% at age 65 to 50% at age 85+. There is less centre variation in life expectancy free of cognitive impairment. Self-perceived health shows less variation with age than the other health domains. As a proportion of the total life expectancy good self-perceived health ranges from 78% in individuals aged 85 and above in Cambridge, to 57% in Nottingham. Compared to men of the same age, women spend a smaller proportion of remaining life free of ill-health, whatever the domain, however, as for the basic prevalence estimates the proportions of life expectancy in good health between the centres varies far more than between the sexes or amongst the age groups (Figure 2). Active life expectancy (free of functional impairment), on the other hand, varied more across age than across centres, with the proportion of remaining life spent active varying from 92% at age 65 to 67% at age 85+ in Gwynedd.

**Figure 3:**
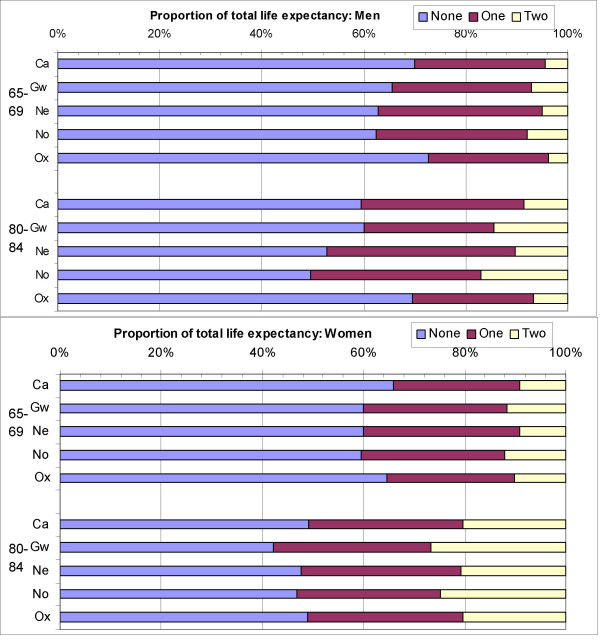
Proportion of life spent by number of impairments for ages 65–69 and 80–84 years and by centre.

As the differences in life expectancy and in the proportions of the population with impairments by centre could contribute to regional differences in impairment burden, we therefore calculated the prevalence of none, one or two (or more) impairments and expressed these as a proportion of remaining life expectancy by age and centre for the two centres with the longest and shortest life. This analysis (not provided in full) revealed no particular pattern in the relationship between the length of life expectancy with one or two impairments and total life expectancy. It can be seen that at all ages (Figure 3), both sexes and in each centre there is a substantial amount of life lived in expectation of impairment particularly at oldest ages.

## Discussion

This five centre study draws on national mortality statistics within local area and is the first to show regional variations in healthy life expectancy at older ages across a range of health domains in England and Wales. Self-perceived health does not show marked variation with age or sex, but does across region. There is considerable regional variation in self-reported functional impairment but not cognitive impairment. The regions with the longest life expectancy have the highest levels of positive self-perceived health, but these do not necessarily appear to be those centres with the lowest impairment burden.

The study provides data from five sites in the early nineties, the sites reflecting the variation that exists nationally. Life expectancies at birth vary across the UK and the regions represented in the study range from lower than expected (Nottingham and Newcastle Standardised Mortality Ratio (SMR) 97 Men and 98 Women) to above the average (Cambridge SMR 103 Men Cambridge, Gwynedd and Oxford 101 Women). Life expectancy for men nationally ranges from 69.7 (Manchester) to 77.9 (East Dorset), and this compares in our study to 71.5 (Newcastle) to 75.7 (Cambridge). Likewise for women nationally the spread is from 75.9 (Eastington, Durham) to 82.6 (East Dorset) with a smaller spread across our regions 77.6 (Newcastle) to 80.3 (Oxford)[32]. Variation in health continues to be of concern and these analyses provide a detailed baseline from which compression or expansion of morbidity with continued population ageing can be ascertained. In addition, if UK government policies that are aimed at reducing inequality are effective this observed variation across site should be reduced over the next decades.

Different measures, such as more objective measures of functional ability and more detailed cognitive tests would produce different absolute results, as would different cutpoints. Since these measures are all closely related it is unlikely that ranking and substantive results would change. There were no systematic differences in the age and sex response rates for this interview, but it is not possible to know whether there were any other systematic differences in nonresponders across centres. The effects of missing data have been explored and the results appear to be robust to different assumptions regarding those people with missing data. Response rates at baseline across the centres were similar, at around 80%.

Self-perceived health is generally accepted as having a very strong relationship to mortality, and is accepted as a more holistic indicator than most others[33,34]. It is of concern and of policy relevance that the centre variation is greater than the variation with age in this measure. Of the three mortality related indicators self-perceived health is the one which is most strongly related to life expectancy according to this analytic method. There does appear to be a longer life expectancy in good self-perceived health in those populations with the longest life expectancy. Such data suggest that the extra years lived by populations in some centres compared to others are years spent in good health and this is not explained by smaller cognitive and functional impairment or morbidity burdens.

Methods used here have been to examine cross-sectional differences and equate those to population estimates of life expectancies. These methods are approximate as they do not follow individuals, however in stable populations they provide robust estimates of healthy life expectancy and are less susceptible to the effects of survey design than longitudinal methods [35]. However they are limited in the extent to which further stratification can be undertaken to adjust for other potential confounders since mortality data at a national level is not available other than by sex, age or area-level. Studies where the unit of analysis has been at the area level have tried to explain variations through ecological correlations with area level socio-demographic[11,18,19], lifestyle behaviours [16-19] and healthcare characteristics[16,17]. Others have explored the relative strength of individual versus area-level characteristics, for instance whether area level of affluence is significantly predictive of self-perceived health when individual level factors are taken account of [36,37]. Socio-demographic and lifestyle factors, particularly low education, unemployment and a high prevalence of smoking have consistently been found to be associated with lower healthy life expectancy at older ages though these may simply be a reflection of deprivation or greater morbidity.

## Conclusion

We have shown the presence of regional differences in impairment and poor health at older ages, in terms of prevalence, and that such differences cannot be explained by the varying disease burden within the centres or by educational level. Moreover differentials in health expectancies were greater between centres than differentials in life expectancy and only in the case of self-perceived health were the extra years lived spent in good health. These suggest that health inequalities in later life still exist and should be addressed by targeting of resources at geographical areas on the basis of health expectancies in order to more appropriately reflect need.

Our analysis suggests that targeting life course effects that have global impact on impairment and health rather than specific disease burden will have the greatest public health benefit.

## Competing interests

The author(s) declare that they have no competing interests.

## Authors' contributions

All authors contributed to writing the paper. FM undertook the final analysis and is guarantor for the paper. LM undertook the original analysis. CJ supervised the work. All authors have seen and agreed the final draft. MRC CFAS is a collaborative study and researchers are acknowledged using corporate authorship.

## Pre-publication history

The pre-publication history for this paper can be accessed here:



## Figures and Tables

**Table 1:** Number of respondents and the proportion with each impairment by age, sex and centre.

	**Cambridge**					**Gwynedd**					**Newcastle**				
	**N**^**†**^	**LE**^**^**^	**S^**# **^%**	**C^**‡ **^%**	F* %	**N**^**†**^	**LE**^**^**^	**S^**# **^%**	**C^**‡ **^%**	F* %	**N**^**†**^	**LE**^**^**^	**S^**# **^%**	**C^**‡ **^%**	F* %
**Men**															
**65–69**	356	14.4	23	1	3	252	13.4	32	1	3	281	11.5	33	1	4
**70–74**	255	11.1	29	1	4	302	10.7	25	3	6	261	8.8	33	2	5
**75–79**	253	8.2	27	4	6	260	7.8	39	6	11	208	7.1	38	2	7
**80–84**	164	5.8	34	4	13	160	5.5	33	5	14	119	5.4	44	5	8
**85+**	79	3.9	22	17	26	86	3.4	33	10	33	61	3.7	43	20	13
**Total**	1107		27	3	7	1060		32	4	9	930		36	3	6
**Women**															
**65–69**	362	17.8	24	1	3	326	17.8	26	1	3	349	15.2	31	1	6
**70–74**	310	14.1	27	1	6	368	14.0	33	2	6	355	11.9	36	1	4
**75–79**	335	10.7	28	3	10	350	10.7	39	4	10	362	9.0	38	3	12
**80–84**	246	7.2	33	10	23	301	7.4	44	11	26	320	6.4	42	16	20
**85+**	241	4.8	34	22	43	223	4.8	41	34	49	208	4.2	35	26	33
**Total**	1494		29	6	14	1568		36	8	15	1594		36	8	13

	**Nottingham**					**Oxford**					**Total**				
	**N**^**†**^	**LE**^**^**^	**S^**# **^%**	**C^**‡ **^%**	F* %	**N**^**†**^	**LE**^**^**^	**S^**# **^%**	**C^**‡ **^%**	F* %	**N**^**†**^	**LE**^**^**^	**S^**# **^%**	**C^**‡ **^%**	F* %

**Men**															
**65–69**	278	13.6	32	1	3	277	14.4	25	1	3	1444	13.2	29	1	3
**70–74**	264	10.8	33	1	5	304	11.0	26	4	2	1386	10.3	29	2	4
**75–79**	236	8.2	38	5	13	203	8.4	27	4	5	1160	7.8	34	4	8
**80–84**	165	6.3	33	6	17	170	5.8	21	5	8	778	5.7	32	5	12
**85+**	66	4.6	43	25	36	100	3.8	24	9	24	392	3.9	31	15	26
**Total**	1009		34	4	10	1054		25	4	5	5160		31	4	8
**Women**															
**65–69**	356	17.0	34	2	7	350	18.2	22	1	5	1743	16.8	28	1	5
**70–74**	333	13.3	29	3	5	397	14.6	29	1	6	1763	13.2	31	2	5
**75–79**	332	10.1	39	4	15	369	11.1	33	3	11	1748	10.0	35	3	11
**80–84**	294	7.2	37	11	25	316	7.9	35	10	23	1477	7.1	38	11	24
**85+**	192	4.8	34	24	51	254	5.3	36	23	39	1118	4.7	36	26	43
**Total**	1507		35	7	17	1686		31	6	14	7846		33	7	15

^†^Total Number, ^^^Life expectancy ^#^Fair/Poorself-perceived health, ^‡^Cognitive impairment, *Functional impairment.

**Table 2:** Logistic regression for self-perceived health and each impairment type.

	Self-perceived Health^†^	Functional Impairment^†^	Cognitive Impairment^†^
Centre:			
Cambridge	1.0 (0.9 – 1.1)	1.0 (0.9 – 1.1)	1.2 (0.9 – 1.4)
Gwynedd	1.5 (1.3 – 1.6)	1.3 (1.1 – 1.4)	1.2 (1.0 – 1.5)
Newcastle	1.4 (1.3 – 1.5)	0.8 (0.7 – 1.0)	1.0 (0.8 – 1.3)
Nottingham	1.2 (1.1 – 1.4)	1.5 (1.2 – 1.7)	1.0 (0.8 – 1.2)
Oxford	1.0 (0.9 – 1.1)	1.0 (0.9 – 1.1)	1.0 (0.8 – 1.2)
Age			
65–69	1.0	1.0	1.0
70–74	1.0 (0.9 – 1.2)	1.1 (0.9 – 1.4)	2.1 (1.3 – 3.5)
75–79	1.1 (1.0 – 1.3)	2.2 (1.8 – 2.8)	3.5 (2.2 – 5.5)
80–84	1.0 (0.9 – 1.1)	4.6 (3.7 – 5.7)	6.4 (4.1 – 10.1)
85+	0.6 (0.5 – 0.7)	12.7 (10.1 – 16.0)	13 (8.3 – 20.4)
Women	1.0 (0.9 – 1.0)	1.7 (1.4 – 1.9)	1.2 (1.0 – 1.5)
Self-perceived health (SPH)	4.4 (3.8 – 5.0)	1.5 (1.2 – 1.9)	1.5 (1.2 – 1.9)
Missing SPH	9.6 (4.6 – 20.0)	2.7 (1.1 – 4.8)	2.7 (1.1 – 4.8)
Functional impairment(FI)	4.2 (3.7 – 4.8)	4.7 (3.7 – 5.9)	4.7 (3.7 – 5.9)
Missing FI	3.0 (1.8 – 4.8)	15.7 (9.2 – 26.8)	15.7 (9.2 – 26.8)
Cognitive impairment (CI)	1.5 (1.2 – 1.8)	4.9 (3.9 – 6.2)	4.9 (3.9 – 6.2)
Missing CI	1.6 (1.0 – 2.6)	6.1 (3.7 – 10.1)	6.1 (3.7 – 10.1)

^†^adjusted for all factors in table, number of health conditions and education
